# Machine Learning in Myasthenia Gravis: A Systematic Review of Prognostic Models and AI-Assisted Clinical Assessments

**DOI:** 10.3390/diagnostics15162044

**Published:** 2025-08-14

**Authors:** Chen-Chih Chung, I-Chieh Wu, Oluwaseun Adebayo Bamodu, Chien-Tai Hong, Hou-Chang Chiu

**Affiliations:** 1 Department of Neurology, Taipei Medical University Shuang Ho Hospital, New Taipei City 235, Taiwan; 10670@s.tmu.edu.tw (C.-C.C.); 21180@s.tmu.edu.tw (I.-C.W.); 2 Department of Neurology, School of Medicine, College of Medicine, Taipei Medical University, Taipei 110, Taiwan; 3 Research & Innovation Center of Artificial Intelligence in Medicine and Health, Shuang Ho Hospital, New Taipei City 235, Taiwan; 4 Taipei Neuroscience Institute, Taipei Medical University Shuang Ho Hospital, New Taipei City 235, Taiwan; 5 Department of Prevention and Community Health, Milken Institute School of Public Health, The George Washington University, Washington, DC 20052, USA; o.bamodu@gwu.edu; 6 Directorate of Postgraduate Studies, School of Clinical Medicine, Muhimbili University of Health and Allied Sciences, Ilala District, Dar es Salaam P.O. Box 65001, Tanzania

**Keywords:** machine learning, myasthenia gravis, myasthenic crisis, prediction, prognosis, systematic review

## Abstract

**Background**: Myasthenia gravis (MG), a chronic autoimmune disorder with variable disease trajectories, presents considerable challenges for clinical stratification and acute care management. This systematic review evaluated machine learning models developed for prognostic assessment in patients with MG. **Methods**: Following PRISMA guidelines, we systematically searched PubMed, Embase, and Scopus for relevant articles published from January 2010 to May 2025. Studies using machine learning techniques to predict MG-related outcomes based on structured or semi-structured clinical variables were included. We extracted data on model targets, algorithmic strategies, input features, validation design, performance metrics, and interpretability methods. The risk of bias was assessed using the Prediction Model Risk of Bias Assessment Tool. **Results**: Eleven studies were included, targeting ICU admission (*n* = 2), myasthenic crisis (*n* = 1), treatment response (*n* = 2), prolonged mechanical ventilation (*n* = 1), hospitalization duration (*n* = 1), symptom subtype clustering (*n* = 1), and artificial intelligence (AI)-assisted examination scoring (*n* = 3). Commonly used algorithms included extreme gradient boosting, random forests, decision trees, multivariate adaptive regression splines, and logistic regression. Reported AUC values ranged from 0.765 to 0.944. Only two studies employed external validation using independent cohorts; others relied on internal cross-validation or repeated holdout. Of the seven prognostic modeling studies, four were rated as having high risk of bias, primarily due to participant selection, predictor handling, and analytical design issues. The remaining four studies focused on unsupervised symptom clustering or AI-assisted examination scoring without predictive modeling components. **Conclusions**: Despite promising performance metrics, constraints in generalizability, validation rigor, and measurement consistency limited their clinical application. Future research should prioritize prospective multicenter studies, dynamic data sharing strategies, standardized outcome definitions, and real-time clinical workflow integration to advance machine learning-based prognostic tools for MG and support improved patient care in acute settings.

## 1. Introduction

Myasthenia gravis (MG) is a chronic, low-prevalence autoimmune neuromuscular disorder characterized by impaired neuromuscular transmission arising from autoantibodies targeting the acetylcholine receptor (AChR) or other components of the neuromuscular junction [1,2]. Clinically, MG manifests as reversible, fluctuating skeletal muscle weakness, affecting extraocular muscles, bulbar muscle groups, and respiratory muscles. The condition may progress from isolated ocular involvement to life-threatening respiratory failure [3]. A subset of patients may experience early and rapid deterioration, advancing to the myasthenic crisis, which necessitates mechanical ventilation and intensive immunotherapy. Approximately 15% to 20% of patients with MG experience a crisis during their lifetime, with mortality rates reaching 4% to 12% in the absence of timely intervention [3,4,5].

Despite the availability of various immunomodulatory and supportive therapies, MG exhibits considerable clinical heterogeneity, with marked interindividual variability in treatment response. Even with standard therapy, some patients experience frequent relapses or rapid deterioration, complicating prognostication [1,6,7]. This unpredictability presents significant challenges for risk stratification, resource allocation, and personalized therapy, creating an urgent need for reliable, data-driven systems to identify high-risk patients and inform care decisions.

Recent advancements in artificial intelligence (AI) have significantly transformed clinical decision making across various medical fields. AI encompasses a broad spectrum of technologies, including expert systems, natural language processing, machine learning, and computer vision. Among these technologies, machine learning has emerged as a compelling risk prediction and disease management tool, demonstrating exceptional utility in addressing complex and heterogeneous medical conditions [8,9,10,11]. Machine learning algorithms offer distinct advantages over traditional statistical methods by processing high-dimensional data, capturing intricate inter-variable relationships, and uncovering hidden prognostic patterns that conventional approaches may fail to detect [12,13,14,15]. The integration of explainability tools, such as SHapley Additive exPlanations (SHAP), has further enhanced transparency and acceptance among clinical stakeholders [10,11,14,16].

In the context of MG, several studies have applied machine learning models to support clinical decision making by predicting outcomes such as intensive care unit (ICU) admission, crisis occurrence, hospitalization duration, and treatment response [17,18,19,20,21,22,23]. Many of these models leverage routinely collected clinical data, including structured features (e.g., demographics, medications, and laboratory results) and semi-structured inputs (e.g., examination videos and smartphone logs) [24,25,26,27], rather than relying on specialized imaging or genomic modalities. However, to our knowledge, no comprehensive systematic reviews have examined this subset of machine learning-based prognostic models in MG. This gap continues to limit their clinical translation and standardization.

In this systematic review and risk-of-bias appraisal, we evaluated machine learning-based prognostic models developed for patients with MG, focusing on those trained using routinely available structured and semi-structured clinical data. To ensure clinical relevance and generalizability, we excluded studies relying primarily on imaging, genomic, or other specialized inputs.

We synthesized model characteristics, including data sources, algorithmic choices, performance metrics, interpretability strategies, and validation methods. Additionally, we performed a structured assessment of bias across included studies. By consolidating current evidence, our review identifies key limitations in existing tools and outlines priorities for future research aimed at developing clinically actionable, personalized prognostic models for MG.

## 2. Materials and Methods

### 2.1. Review Protocol and Registration

This systematic review adhered to the 2020 Preferred Reporting Items for Systematic Reviews and Meta-Analyses (PRISMA) guidelines [28]. A completed PRISMA checklist is available in Supplementary Table S1. The study protocol was developed before the initiation of the review and included predefined objectives, article selection criteria, search strategies, data extraction procedures, and risk-of-bias assessment methods. The protocol was prospectively registered in the International Prospective Register of Systematic Reviews (PROSPERO) database (registration number: CRD420251026876), available at https://www.crd.york.ac.uk/PROSPERO/view/CRD420251026876 (accessed on 8 June 2025).

### 2.2. Eligibility Criteria

Studies were included if they (1) included patients diagnosed with MG; (2) applied machine learning algorithms or AI algorithms for predictive model development, validation, or automated clinical assessment; (3) assessed at least one clinical outcome or relevant physiological feature linked to disease progressions, such as exacerbation, hospitalization, or myasthenic crisis; and (4) utilized structured clinical or semi-structured observational data as input features, including demographic information, antibody status, disease severity, medication use, or laboratory results.

To ensure clinical applicability and focus on prognostic models based on routinely accessible clinical data, we deliberately excluded studies under the following conditions: (1) studies relying primarily on imaging data (e.g., magnetic resonance imaging, computed tomography, or positron emission tomography), genomic data, or electromyography; (2) studies that employed only conventional statistical methods (e.g., Cox regression or logistic regression without machine learning integration); (3) studies that focused exclusively on diagnostic classification without contributing to disease monitoring or prognostic inference; and (4) non-original research articles, including conference abstracts, commentaries, editorials, case reports, or review articles.

The exclusion of imaging, genomic, and electrophysiological studies intentionally prioritized models that can be readily applied in real-world clinical settings using easily obtainable data without specialized equipment or advanced processing techniques. This design choice aligns with our goal to promote practical and widely implementable machine learning solutions for MG prognostication.

### 2.3. Data Source and Search Strategy

PubMed, Embase, and Scopus were comprehensively searched to identify relevant studies published between 1 January 2010 and 31 May 2025. The search strategy was tailored to each database using controlled vocabulary (e.g., MeSH and Emtree terms) and keyword combinations related to myasthenia gravis, machine learning, artificial intelligence, and specific supervised and unsupervised algorithms, including support vector machines (SVMs), neural networks, random forests, extreme gradient boosting (XGBoost), linear discriminant analysis (LDA), soft independent modeling of class analogy (SIMCA), and chemometric approaches. The search was restricted to English-language publications. All retrieved records were imported into reference management software (EndNote 20, Clarivate Analytics, Philadelphia, PA, USA), and duplicates were removed before screening. The full search strategy for each database is presented in Supplementary Table S2.

### 2.4. Article Selection

Two independent reviewers screened all titles and abstracts retrieved from the database searches. Full-text articles were obtained and reviewed for studies meeting the inclusion criteria or when eligibility could not be determined from the abstract alone. Discrepancies were resolved through discussion until a consensus was reached. Studies meeting all predefined criteria were included in the analysis.

### 2.5. Data Extraction

The following data were extracted from each included study: first author, publication year, study design, sample size, machine learning approach, predicted outcomes, input variables, performance metrics, external validation status, and explainability method use. All data were recorded in a standardized spreadsheet. Additional data, such as information on cross-validation, calibration, and specific techniques (e.g., SHAP and feature importance rankings), were collected.

### 2.6. Risk-of-Bias and Quality Assessments

Risk of bias was systematically evaluated using the Prediction Model Risk of Bias Assessment Tool (PROBAST) across four domains: participants, predictors, outcome definition, and statistical analysis [29]. Two researchers independently conducted the assessments. Between-reviewer discrepancies were resolved through discussion, with arbitration by a third reviewer when necessary. All studies were included in the analysis. The results were used to interpret model performance and evaluate evidence strength.

### 2.7. Data Synthesis and Analysis

All extracted data were organized using a standardized format and reviewed narratively for structured comparison. Given the substantial heterogeneity observed across the included studies in designs, data sources, outcome definitions, population characteristics, and modeling approaches, a quantitative meta-analysis was not feasible. Instead, a thematic classification based on prediction targets and algorithm types was performed. Performance metrics and interpretability methods were compared among the studies to comprehensively assess clinical relevance and transparency.

## 3. Results

### 3.1. Review Sample

**Figure 1:**
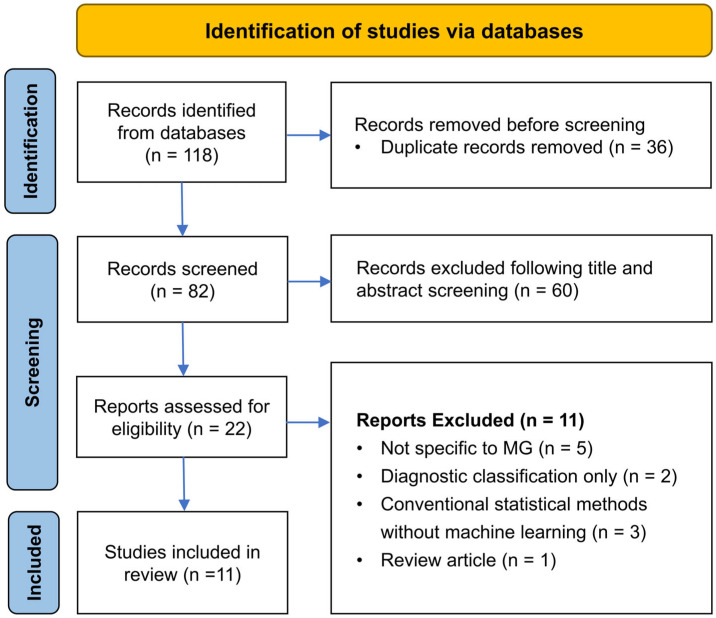
PRISMA flow diagram of study selection process.

The systematic search identified a total of 118 records across three major databases (PubMed: 22, Embase: 52, and Scopus: 44). After the removal of 36 duplicates, 82 records were subjected to title and abstract screening. Of these records, 60 were excluded based on predefined eligibility criteria. The remaining 22 articles underwent full-text review, resulting in the exclusion of 11 studies for the following reasons: lack of relevance to MG (*n* = 5), emphasis solely on diagnostic modeling (*n* = 2), reliance on conventional statistical methods without machine learning integration (*n* = 3), and classification as non-original research (*n* = 1). Ultimately, 11 studies met the inclusion criteria and were thus included in the final systematic review. The selection process is detailed in Figure 1.

### 3.2. Study Characteristics

This systematic review included 11 original articles published between 2022 and 2025 (Table 1). Of these, seven developed predictive models while four centered on clustering or automated scoring strategies. The study cohorts comprised 51 to 890 patients with MG. All studies employed structured clinical or semi-structured observational data as input features—for example, demographic characteristics, autoantibody status, Myasthenia Gravis Foundation of America (MGFA) classification, comorbidities, medication use, laboratory values, and digital monitoring logs. Several studies incorporated novel data modalities, such as repeated video recordings or smartphone-based digital phenotyping. Four studies implemented prospective or pseudo-prospective designs, while the remaining were retrospective. Three studies integrated AI pipelines into telemedicine-based Myasthenia Gravis Core Examination (MG-CE) video examinations. One study developed a smartphone-based digital diary system for longitudinal symptom monitoring, while others focused on predicting clinical outcomes such as ICU admission, hospital stay length, myasthenic crisis, or prolonged mechanical ventilation.

Regarding geographic distribution, five studies were conducted in Asia (three in Taiwan and two in China), three in the United States, and three in Europe (two in Germany, one in the Netherlands). All studies applied machine learning or AI algorithms to classify patients or predict clinical trajectories, with performance evaluated using cross-validation, external validation cohorts, or inter-rater reliability comparisons. While some studies incorporated interpretability frameworks (e.g., SHAP or decision rules), others focused on feasibility and algorithm development without formal performance benchmarking.

### 3.3. Model Characteristics and Performance

Of the 11 included studies, 7 developed predictive models for clinical outcomes. Among these, the highest-performing models were the C5.0 decision tree, random forest, XGBoost, multivariate adaptive regression splines (MARS), and k-means clustering. Six studies employed classification algorithms, with the area under the receiver operating characteristic curve (AUC) used as the primary metric for evaluating model performance. The highest performance was exhibited by a model developed by Xu et al. [21], an XGBoost framework for predicting 6-month post-intervention status (PIS) in AChR antibody-positive patients with generalized MG. This model achieved AUC values of 0.944 and 0.908 in internal and external validation tests. Kuo et al. [18] also used the XGBoost algorithm to develop a model for predicting ICU admission; their model achieved an AUC value of 0.894 in cross-validation tests. Chang et al. [17] used C5.0 decision trees to anticipate the need for ICU admission; their model achieved an AUC value of 0.814. Zhong et al. [19] used a random forest model to differentiate between patients based on treatment response; this model achieved AUC values of 0.84, 0.74, and 0.79. Bershan et al. [22] used a random forest model to identify patients at increased risk of crisis; their model achieved an AUC value of 0.765. Heider et al. [23] used logistic regression to identify risk factors for prolonged mechanical ventilation during myasthenic crisis, yielding a cross-validated AUC of 0.78.

Only Chang et al. [20] analyzed a continuous outcome using MARS to predict hospitalization duration. Their reported error metrics included a mean absolute percentage error of 0.524, a symmetric mean absolute percentage error of 0.409, and a relative absolute error of 1.133. Steyaert et al. [24] used unsupervised learning techniques, combining principal component analysis with *k*-means clustering, to perform exploratory digital phenotyping and identify patient subtypes.

Among the seven prognostic modeling studies, only two (Xu et al. and Zhong et al.) [19,21] performed external validation using independent cohorts, while the remaining five relied on internal validation methods such as cross-validation or dataset splitting. Kuo et al. and Heider et al. [18,23] presented comprehensive information on calibration analyses, with Kuo using calibration plots and Brier scores, and Heider et al. utilizing the Generalized Unbiased Evaluation of Scoring Systems (GUESS) algorithm for probability calibration. Four studies used SHAP values for interpretability analysis (Table 1).

Distinct from the predictive modeling studies, four investigations explored either unsupervised clustering or AI-assisted clinical assessments. Steyaert et al. [24] applied k-means clustering with principal component analysis (PCA) visualization to stratify symptom fluctuation subtypes using smartphone-based monitoring. The remaining three studies (Lesport et al. [25]; Garbey et al. [26,27]) developed automated MG-CE scoring pipelines leveraging computer vision and natural language processing techniques. Although predictive performance was not evaluated, one study reported up to 25% inter-rater variability, underscoring the potential of AI-based scoring to enhance clinical consistency [27].

### 3.4. Risk of Bias and Applicability

We used PROBAST to assess study quality and risk of bias across four domains: participants, predictors, outcome definitions, and statistical analysis. Only the studies by Zhong et al. [19], Xu et al. [21], and Heider et al. [23] were rated as having low risks across all domains, whereas the remaining studies exhibited high risks in two or more domains.

The risk of bias was predominantly concentrated in the predictor and analysis domains. Common methodological limitations identified by Chang et al. [5,6], Kuo et al. [18], Steyaert et al. [24], and Bershan et al. [22] included insufficient sample size, class imbalance, lack of external validation or calibration analysis, and temporal confounding between input variables and outcomes, which introduced a risk of information bias. The study by Steyaert et al. [24] diverged from conventional model development; the researchers used daily self-reported functional deterioration status as a prognostic variable and combined it with MG-affected activities of daily living scores and symptom clustering for feature analysis. Despite being conceptually innovative, the study mentioned above was primarily exploratory in nature, lacking clinical intervention or validation through standard event-based outcomes. Its methodological characteristics differed from those of the other studies we reviewed, thereby warranting contextual interpretation of its assessment results according to the research objectives. By contrast, Xu et al. [21], Zhong et al. [19], and Heider et al. [23] applied well-defined inclusion criteria and structured input selection, used clinically relevant outcome definitions, and incorporated internal or external validation tests, partially mitigating limitations inherent to retrospective designs.

In terms of applicability, although the majority of studies relied on clinical data, many encountered implementation barriers attributable to restricted data sources, insufficient variable coverage, and undefined deployment scenarios. The studies by Xu et al. [21], Zhong et al. [19], and Heider et al. [23] exhibited alignment between data composition, model structure, and target populations, indicating increased feasibility for integration into real-world settings. Despite being published as a Letter to the Editor, the study by Heider et al. [23] met our inclusion criteria with formal model development, multicenter data, and validation results and was therefore included.

Three studies by Lesport et al. [25] and Garbey et al. [26,27] focused on AI-assisted video-based scoring of neurological examinations, specifically the MG-CE and MG-ADL scales. These studies aimed to demonstrate technical feasibility and inter-rater consistency rather than clinical outcome prediction. While methodologically innovative, their reliance on audiovisual input features and absence of comparator models highlight the need for further validation in diverse clinical contexts.

A detailed summary is provided in Table 2.

**Figure 2:**
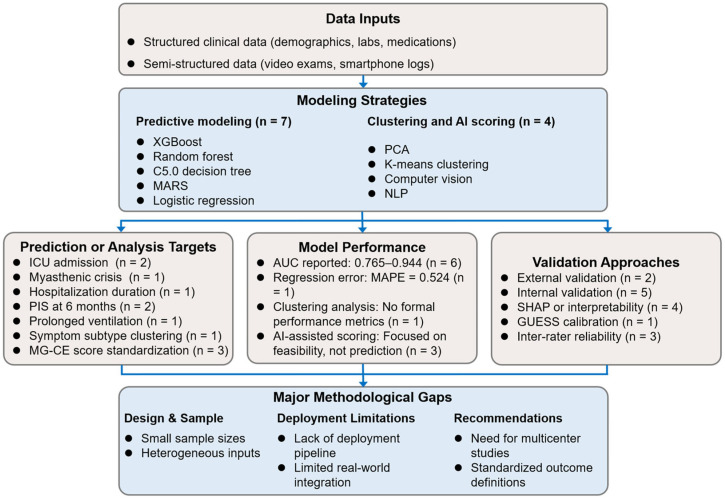
Synthesis of data types, model strategies, outcome targets, and methodological considerations across included studies. AUC, area under the receiver operating characteristic curve; ICU, intensive care unit; MAPE, mean absolute percentage error; MARS, multivariate adaptive regression splines; MG-CE, Myasthenia Gravis Core Examination; NLP, natural language processing; PCA, principal component analysis; PIS, post-intervention status; SHAP, SHapley Additive exPlanations; XGBoost, eXtreme Gradient Boosting.

Collectively, the included studies predominantly employed ensemble and decision tree-based algorithms, with ICU admission and short-term functional outcomes being the most frequently targeted endpoints. However, substantial heterogeneity was observed in data sources, sample sizes, model architectures, and validation strategies, underscoring the fragmented and exploratory nature of current MG prognostic research. In response, a comprehensive conceptual framework was developed to synthesize data sources, machine learning methodologies, prediction targets, validation strategies, and principal methodological limitations across the included studies (Figure 2). This framework visually integrates the analytical workflow and key findings of this systematic review, providing a structured overview for future research development.

## 4. Discussion

This systematic review synthesized current evidence on machine learning and AI applications in MG, encompassing prognostic modeling and automated clinical assessment. While machine learning applications in MG remain nascent, our findings indicate that these models demonstrate potential for supporting clinical decision making, particularly in predicting adverse outcomes, including disease deterioration, hospitalization, and myasthenic crisis.

Most studies centered on prognostic modeling using structured, routinely collected clinical data—such as demographics, MGFA classification, and laboratory results—highlighting a focus on practical, implementation-ready inputs. A smaller subset explored non-predictive uses of AI, including automated examination scoring and symptom-based patient subtyping. These studies reflect a broadening interest in integrating AI into various stages of MG care, from early risk prediction to standardized assessment and remote monitoring.

While AI encompasses a broad spectrum of technologies, this review concentrated on machine learning applications with low technical barriers to adoption. Studies relying primarily on high-cost or specialized modalities—such as imaging for anatomical assessment, genomics, or electrophysiology—were excluded. For example, purely imaging-based AI applications in MG have mainly focused on thymoma detection or classification, often involving non-MG patients and offering limited prognostic utility [30,31,32]. In contrast, we prioritized models based on clinically pragmatic input sources with higher translational relevance and broader potential for deployment across healthcare settings.

Despite favorable performance metrics (AUC: 0.765–0.944), considerable methodological heterogeneity was observed. Only three studies [19,21,23] demonstrated low risk of bias across all PROBAST domains, reflecting greater analytical rigor and potential generalizability. Single-center designs, small sample sizes, lack of calibration assessment, and minimal deployment planning limited the remaining studies. While some employed interpretability techniques to enhance clinical relevance, few addressed execution-phase concerns such as workflow integration, model usability, or implementation logistics. None of the included models demonstrated readiness for validated deployment in routine practice.

A persistent barrier to progress in this field is the lack of large, diverse, and openly accessible clinical datasets. A total of 6 of the 11 studies included in this review were derived from just two small and overlapping sources. Three studies [17,18,20] were conducted using data from the same single-center registry in Taiwan, resulting in substantial duplication of patient cohorts and input variables. Similarly, three studies analyzing AI-assisted quantification of MG-CE and MG-ADL used the same video dataset [25,26,27]. Despite differing research aims, these studies were not based on independent data collections. This limited data diversity constrains the variability of model training inputs and the assessment of model performance across different clinical settings and patient populations. Moreover, reusing the same cohort across multiple publications risks overstating evidence strength and can misrepresent the field’s maturity. These findings underscore a structural bottleneck in MG AI research: the urgent need for collaborative multicenter efforts to build broader, demographically inclusive datasets. Advancing the development and accessibility of such repositories will be essential for enabling robust, generalizable, and clinically deployable AI applications in MG.

### 4.1. Targeted Outcomes and Their Relevance to Critical Care

Several targeted outcomes were directly related to acute care triage and resource allocation in the included studies. Chang et al. and Kuo et al. used the C5.0 decision tree and XGBoost algorithms, respectively, to develop models for predicting the risk of ICU admission [17,18]. Both groups identified MGFA classification and disease duration as significant predictors. The model developed by Kuo et al. [18] achieved an AUC value of 0.894 and revealed a nonlinear effect of age and the influence of thymectomy status through SHAP analysis. In their pseudoprospective study, Bershan et al. [22] used a random forest classifier to estimate myasthenic crisis; they highlighted serum creatinine fluctuations and interunit transfers as key indicators. Xu et al. [21] constructed a rigorously validated framework for determining 6-month MGFA-PIS for AChR antibody-positive patients with generalized MG. The framework incorporated inflammatory indices, such as the systemic immune-inflammation index, neutrophil-to-lymphocyte ratio, platelet-to-lymphocyte ratio, and quantitative MG scores, highlighting the complementary utility of biological markers in personalized prognostication. Using a multicenter dataset and Ensemble Feature Selection, Heider et al. [23] developed a logistic regression model to predict prolonged mechanical ventilation (>15 days) during myasthenic crisis. Their model, calibrated using GUESS, achieved an AUC of 0.78 and incorporated clinically accessible predictors such as age, MGFA classification, comorbidities, pneumonia, delirium, and cardiopulmonary resuscitation. In a secondary analysis, Chang et al. [20] used MARS to estimate hospitalization duration, the only continuous outcome assessed in our review. Steyaert et al. [24] conducted a prospective digital phenotyping investigation combining electronic patient-reported outcomes with wearable sensor data. They used k-means clustering to identify symptom fluctuation patterns. Despite being exploratory, this approach showcases the integration of remote monitoring with unsupervised learning in the management of MG.

### 4.2. Balancing Transparency and Accuracy in Algorithm Design

The tension between algorithmic performance and interpretability remains crucial in MG prognostic modeling. Chang et al. [17] used C5.0 decision trees to create explicit clinical rules with visualization capabilities suitable for rapid clinical judgment. Kuo et al. adopted a gradient-boosting approach paired with SHAP to explain complex relationships, achieving higher accuracy at the cost of higher logical complexity [16]. Heider et al. [23] demonstrated logistic regression combined with GUESS-based calibration, balancing predictive performance with transparency through clinically grounded variables and probability refinement techniques. Rule-based systems offer advantages in comprehensibility and adoption flexibility but struggle with high-dimensional data and nonlinear interactions. Ensemble approaches overcome these limitations but require interpretive mechanisms to fulfill clinical and operational needs [14,16,33]. Model architecture should be selected according to task characteristics and application scenarios rather than relying solely on performance metrics or algorithmic form. Outside the scope of prognostic modeling, automation-oriented studies by Lesport and Garbey [25,26,27] adopted computer vision and natural language processing pipelines, underscoring the interpretability limitations of AI systems applied beyond traditional prognostic modeling.

In acute care settings, clarity and workflow compatibility often outweigh marginal gains in predictive power. By contrast, long-term management and personalized treatment benefit more from systems that can handle layered clinical and behavioral inputs and support nuanced patient stratification [12,34]. Embedding domain-specific considerations and task-oriented design principles during early model development phases can substantially enhance clinical relevance and implementation feasibility.

### 4.3. Bridging the Gaps Between Explainability and Clinical Acceptability

Four studies used interpretation techniques such as SHAP values (Kuo et al., Xu et al., and Bershan et al. [18,21,22]) and decision tree rules (Chang et al. [17]). These methods enhance result visualization and variable interpretation transparency, addressing explainability needs in clinical AI applications [33,34,35]. However, the maturity of interpretation techniques does not necessarily translate to accurate understanding and practical application by clinical teams. Xu et al. identified inflammatory indicators as key prognostic factors. Kuo et al. highlighted the effect of age on MGFA classification in ICU triage. However, graphs depicting feature contribution can illustrate model logic; the knowledge required for interpreting graphical outputs may impede clinical adoption [16,36]. Bershan et al. conducted a SHAP analysis but did not define trigger conditions or operational guidelines. This suggests that most explanatory modules remain focused on post hoc analysis rather than supporting real-time decision making [12,36].

Our review indicates that interpretation techniques should be designed synchronously with clinical workflows, such as setting trigger points, standardizing output formats, and mapping clinical terminologies, to improve usability and integration into decision-making processes.

### 4.4. The Blurred Boundary Between Prediction and Early Warning

The included studies primarily focused on risk assessment, with model performance evaluated using metrics such as the AUC value. Once applied in clinical practice, risk assessment models often shift from retrospective analyses to real-time alert mechanisms designed to flag high-risk individuals and trigger timely interventions [34]. This shift involves reconstructing the fundamental nature of the task: prediction focuses on long-term performance and accuracy, whereas early warning requires explicit definitions of thresholds, output frequency, and decision-triggering processes [33,34]. Bershan et al. used routine clinical data to stratify patients based on the risk of severe exacerbation; however, the approach remained a proof of concept without integrating real-time monitoring or response protocols. Similarly, Chang et al. and Kuo et al. [17,18] developed ICU-focused models but did not specify how outputs were incorporated into frontline decision-making processes, such as rapid response team activation or ICU assessment initiation. As machine learning tools transition from retrospective analysis to real-time decision support, their effectiveness depends not only on algorithmic precision but also on seamless integration into clinical workflows, translating outputs into actionable signals [14,33,34].

### 4.5. Critical Care Applications and Implementation Challenges

Machine learning methods often outperform traditional statistical approaches by capturing complex patterns in high-dimensional clinical, laboratory, and physiological data, enabling tailored assessments in acute neuromuscular conditions such as myasthenic crises [37,38,39,40]. These approaches leverage diverse clinical, laboratory, and physiological inputs, facilitating risk stratification and optimizing intervention timing during episodes of severe neuromuscular deterioration. Despite these findings, the relationship between algorithm-generated output and clinical accountability remains unclear. Most included studies reported classification probabilities or risk assessments without explicitly stating whether these outputs were intended to serve as reference recommendations or as determinants for resource allocation and care decisions. In terms of ICU admission [17,18], the substantive effect of high-accuracy risk assessment on care level depends on whether outputs can be rapidly interpreted, incorporated into current decision frameworks, and interfaced with standardized clinical trigger points. Without operational guidelines and response protocols, even highly accurate systems may remain disconnected from care processes and raise questions regarding accountability and patient rights. Zhong et al. [19] used a multicenter random forest model as an online, real-time prediction platform, marking a rare example of preliminary clinical deployment. Bershan et al. [22] published their complete analysis code, becoming the only study with full reproducibility and open-source characteristics, demonstrating a commitment to transparency. Heider et al. [23] also developed a web-based interface (POLAR) to support probability estimation for prolonged mechanical ventilation, which may assist in clinical decision making pending further prospective validation and workflow integration.

Although improving analytical accuracy is essential, real-world use demands clearly defined integration points, operational clarity, and shared responsibility between algorithm developers and clinical teams.

### 4.6. Research Limitations and Future Directions

Despite promising results, current machine learning models face substantial barriers to clinical integration and demonstrate insufficient methodological rigor.

First, we excluded data modalities not routinely leveraged for prognostic decision making in MG to enhance generalizability. Studies utilizing genetic analyses or specialized diagnostics were omitted. While this ensures broad applicability, it may exclude complementary prognostic insights. Additionally, AI studies in MG utilizing imaging predominantly focus on thymoma detection rather than prognostic prediction, often restricted to thymoma subgroups and potentially including non-MG patients, limiting generalizability. Thus, excluding primarily imaging-based studies was pragmatic and aligned with our focus on broad, subtype-inclusive prognostic modeling.

Second, most included studies were retrospective, single-center investigations with limited sample sizes. Only two studies performed external validation using multicenter data. Six of the eleven studies utilized only two overlapping data sources, constraining training variability and raising generalizability concerns. This reflects the broader challenge of scarce, high-quality multicenter datasets. Future research requires expanded data-sharing initiatives and collaborative infrastructure to improve model robustness and real-world applicability.

Third, although several models applied interpretability tools such as SHAP or decision trees, these remained disconnected from clinical workflows. Most lacked real-time interfaces, operational triggers, or integration into routine decision-making processes, limiting their impact on actual care delivery [34,36].

Fourth, existing models emphasize acute outcomes (ICU admission, mechanical ventilation) and rely heavily on static, clinician-reported variables. This approach limits their ability to capture the temporal and individualized nature of MG progression. Intermediate and patient-centered endpoints, such as long-term functional status, quality of life, and rehospitalization, remain understudied. Although few studies incorporated time-series data, advanced sequence-based architectures (recurrent neural networks, Transformers) remain underexplored [41,42]. Additionally, patient-reported parameters including fatigue, emotional distress, and sleep quality are rarely included, despite their potential value for prognostic assessment [36,43,44,45].

It is worth noting that an increasing body of recent research has applied novel machine learning techniques to diverse aspects of MG, including infrared spectral imaging [46], facial recognition-based assessment [47], multi-omics profiling (e.g., metabolomics, microbiome, genomics) [48,49,50,51], and health economic modeling [52]. While methodologically innovative, these studies were excluded as the scope of this review focused on prognostic models built on routinely available structured clinical data, whereas most targeted diagnosis, multi-omics biomarker discovery, or cost prediction, and often relied on non-structured inputs. Despite being outside our inclusion criteria, these research directions hold substantial potential. As clinical workflows increasingly integrate sensor-based technologies, multi-omics analyses, and multimodal data fusion, such models may become integral to MG monitoring and personalized care. Continued AI advancements will likely enable hybrid frameworks that combine structured clinical data with richer, non-traditional inputs, offering new opportunities for comprehensive prognostic modeling in MG.

Despite these limitations, the scarcity of machine learning studies utilizing structured clinical data for MG prognostic modeling underscores this systematic review’s unique contribution. Our findings provide a foundational framework for future research and may guide emerging model development while supporting the integration of diverse clinical data sources for eventual implementation.

In summary, MG presents clear diagnostic criteria, quantifiable outcomes, and variable disease trajectories, positioning it as an optimal candidate for artificial intelligence-driven precision neuroimmunology. Through continued integration of high-quality data repositories and advanced algorithms, intelligent systems may become fundamental components for predicting disease exacerbations, treatment responses, and care optimization, ultimately improving clinical outcomes for MG patients.

## 5. Conclusions

This systematic review comprehensively evaluated machine learning models for prognostication in MG, providing detailed insights into predictive targets, methodologies, and application readiness based on structured and semi-structured clinical data. The 11 studies assessed ICU admission, myasthenic crisis, treatment response, hospitalization duration, symptom fluctuations, and AI-assisted clinical scoring. Prognostic models showed potential for acute care applications, especially in risk stratification and resource allocation, while non-predictive studies illustrated opportunities for standardizing neurological assessments. However, substantial methodological variability was observed across studies. Common limitations included reliance on retrospective single-center data, insufficient calibration reporting, limited external validation, and a lack of fairness assessments. Additionally, few studies provided interfaces or pathways for real-time clinical integration, limiting their practical deployment.

This review introduces a comparative framework highlighting heterogeneity in prediction targets, data sources, algorithmic strategies, and validation approaches across current MG AI studies. It underscores critical gaps in deployment readiness, including trade-offs between model complexity and interpretability, and the absence of early warning integration and fairness considerations. These insights extend beyond MG, providing an evidence-based roadmap for developing adaptable tools that combine real-world utility with methodological rigor, particularly in critical care environments where timely, responsible implementation is essential.

Future studies should prioritize multicenter collaboration, dynamic data pipelines, and clear implementation pathways to enhance the clinical interpretability and usability of predictive tools. As data accessibility and algorithmic transparency advance, machine learning holds strong potential for improving risk assessment and enabling timely, personalized care in patients with MG.

## Figures and Tables

**Table 1:** Summary of included studies for myasthenia gravis prognostication using structured clinical data.

Author (Year) [Ref.]	Study Design	Region/Center Type	Sample Size	Prediction Target	No. of Input Features	Representative Features	Best-Performing Model (Comparators)	Validation Strategy	Performance	Interpretability Method	Remarks
Chang et al. (2022) [17]	Retrospective	Taiwan/Single-center	228	ICU admission	20	MGFA, thymoma, azathioprine, disease duration, sex, onset age	Decision Tree C5.0 (CART, C4.5, LR)	Cross-validation (10-fold)	AUC 0.814	Decision rules	ICU admission rules via decision tree
Kuo et al. (2024) [18]	Retrospective	Taiwan/Single-center	314	ICU admission	14	MGFA, thymectomy, disease duration, age	XGBoost (LR, SVM, random forest)	Cross-validation, calibration (10-fold)	AUC 0.894	SHAP	Calibration plot, Brier score
Zhong et al. (2023) [19]	Retrospective	China/Multicenter	890	PIS at 6-month	25	QMG: left arm outstretch, corticosteroid, QMG: ptosis, antibody	Random Forest	External validation (independent cohort)	AUC 0.84 (improved), 0.74 (unchanged), 0.79 (worse)	SHAP	Online prediction tool
Chang et al. (2023) [20]	Retrospective	Taiwan/Single-center	196	Length of hospital stay	18	Disease duration, age, MGFA, daily prednisolone dose	MARS (Lasso MLR, CART, random forest)	Cross-validation (10-fold)	MAPE: 0.524, SMAPE: 0.409, RAE: 1.133	Variable thresholds	Continuous outcome
Xu et al. (2024) [21]	Retrospective	China/Multicenter	202	PIS at 6-month	8	SII, NLR, disease duration, PLR, QMG score	XGBoost (LR, SVM, random forest)	External validation (independent cohort)	AUC 0.944 (internal) AUC 0.908 (external)	SHAP	AChR-Ab+ subgroup
Steyaert et al. (2023) [24]	Prospective observational	USA/Decentralized virtual study (multi-state)	82	Symptom exacerbation subtype clustering	40 (18 static, 22 longitudinal)	Daily step count, MG-ADL symptom scores, medication group, time since diagnosis	K-means clustering	Cluster selection via elbow method	N/A (unsupervised; not benchmarked against outcomes)	Cluster profiling via random forest; PCA visualization	Smartphone-based digital phenotyping
Bershan et al. (2025) [22]	Pseudo-prospective	Germany/Single-center	51	Myasthenic crisis	79	Creatinine trend, lymphocyte variability, hospitalization trajectory	Random forest (Lasso regression)	Repeated holdout (100 runs)	AUC 0.765	Feature stability, contribution maps	Open-source code
Heider et al. (2024) [23]	Retrospective	Germany/Multicenter	195	Prolonged mechanical ventilation (>15 days)	9 (after Ensemble Feature Selection)	Age, comorbidities, late-onset MG, MGFA IVb, delirium, pneumonia, CPR	Logistic regression (no comparator)	10 × 10-fold cross-validation	AUC 0.78	Ensemble Feature Selection	Web-based prediction tool (POLAR)
Lesport et al. (2024) [25]	Algorithm development	USA/Single center	51	MG-CE scoring automation via AI	Not reported	Eye/body motion, NLP-based vocalization features	AI-based pipeline (no comparator)	Not reported	No formal metric reported	None	Proposed telemedicine scoring enhancement
Garbey et al. (2024) [26]	Prospective observational	USA/Single center	51 MG, 15 controls	AI-assisted MG-CE quantification	Not reported	Lid and eye position, arm movement, breath count, vocalization/NLP	Custom AI pipeline (Computer Vision + NLP)	Repeated video recordings on separate days	No formal metric reported	None	Cheek puff limited; lighting and camera angle issues
Garbey et al. (2025) [27]	Prospective observational	USA/Single center	51	Reproducibility and variability in MG-CE and MG-ADL	Not reported	Eye motion, speech, examiner instruction	AI-based analysis pipeline (no comparator)	Inter-rater comparison	Up to 25% scoring variation	None	Variability attributed to instruction and technical limitations

Note: Representative features reflect key variables highlighted by authors or identified via interpretability techniques (e.g., SHAP, decision rules). Abbreviations: AChR-Ab, acetylcholine receptor antibody; ADL, activities of daily living; AUC, area under the receiver operating characteristic curve; CART, classification and regression tree; CPR, cardiopulmonary resuscitation; ICU, intensive care unit; LR, logistic regression; MAPE, mean absolute percentage error; MARS, multivariate adaptive regression splines; MG-CE, Myasthenia Gravis Core Examination; MGFA, Myasthenia Gravis Foundation of America; MLR, multiple linear regression; NLP, natural language processing; NLR, neutrophil-to-lymphocyte ratio; PCA, principal component analysis; PIS, post-intervention status; PLR, platelet-to-lymphocyte ratio; QMG, quantitative myasthenia gravis; RAE, relative absolute error; SHAP, SHapley Additive exPlanations; SII, systemic immune-inflammation index; SMAPE, symmetric mean absolute percentage error; SVM, support vector machine; XGBoost, eXtreme gradient boosting.

**Table 2:** Risk of bias and applicability assessment using PROBAST.

Study (First Author, Year [Ref.])	Participants	Predictors	Outcome	Analysis	Overall Risk of Bias	Applicability Concerns
Chang, 2022 [17]	High	High	Low	High	High	High
Kuo, 2024 [18]	High	Low	Low	High	High	High
Zhong, 2023 [19]	Low	Low	Low	Low	Low	Low
Chang, 2023 [20]	High	High	High	High	High	High
Xu, 2024 [21]	Low	Low	Low	Low	Low	Low
Steyaert, 2023 [24]	High	High	Not Applicable	High	High	High
Bershan, 2025 [22]	High	High	Low	High	High	High
Heider, 2024 [23]	Low	Low	Low	Low	Low	Low
Lesport, 2024 [25]	High	High	Not Applicable	High	High	High
Garbey, 2024 [26]	High	High	Not Applicable	High	High	High
Garbey, 2025 [27]	High	High	Not Applicable	High	High	High

Note: Risk of bias was assessed across four domains according to PROBAST: participants, predictors, outcomes, and analysis. Applicability concerns were evaluated globally. Risk levels were categorized as Low or High. The Outcome domain was marked as Not Applicable for studies involving exploratory symptom-based clustering without predefined clinical endpoints. Two reviewers conducted all assessments independently following PROBAST guidelines, with discrepancies resolved through discussion. Abbreviation: PROBAST, Prediction model Risk of Bias Assessment Tool.

## Data Availability

All data utilized in this systematic review were derived exclusively from previously published studies available in the public domain. Data were extracted from peer-reviewed articles indexed in major databases, including PubMed, Embase, and Scopus. No new datasets were generated or collected since this review is based solely on secondary data. Consequently, data sharing is not applicable.

## Supplementary Materials

The following supporting information can be downloaded at https://www.mdpi.com/article/10.3390/diagnostics15162044/s1, Supplementary Table S1. PRISMA 2020 Checklist; Supplementary Table S2. Search strategy for each database.

## Abbreviations

The following abbreviations are used in this manuscript:

AChR	Acetylcholine receptor
ADL	Activities of daily living
AUC	Area under the curve
AI	Artificial intelligence
CART	Classification and regression tree
ICU	Intensive care unit
LR	Logistic regression
MAPE	Mean absolute percentage error
MARS	Multivariate adaptive regression splines
MG	Myasthenia gravis
MG-CE	Myasthenia Gravis Core Examination
MGFA	Myasthenia Gravis Foundation of America
MLR	Multiple linear regression
NLP	Natural language processing
NLR	Neutrophil-to-lymphocyte ratio
PCA	Principal component analysis
PIS	Postintervention status
PLR	Platelet-to-lymphocyte ratio
QMG	Quantitative myasthenia gravis
RAE	Relative absolute error
SHAP	SHapley Additive exPlanations
SII	Systemic immune-inflammation index
SMAPE	Symmetric mean absolute percentage error
SVM	Support vector machine
XGBoost	eXtreme gradient boosting
